# Health-Promoting Compounds in Stevia: The Effect of Mycorrhizal Symbiosis, Phosphorus Supply and Harvest Time

**DOI:** 10.3390/molecules25225399

**Published:** 2020-11-18

**Authors:** Silvia Tavarini, Clarissa Clemente, Cecilia Bender, Luciana G. Angelini

**Affiliations:** 1 Department of Agriculture, Food and Environment, University of Pisa, Via del Borghetto 80, 56124 Pisa, Italy; silvia.tavarini@unipi.it (S.T.); clarissa.clemente@agr.unipi.it (C.C.); 2 NUTRAFOOD Interdepartmental Research Center, Nutraceuticals and Food for Health, University of Pisa, 56124 Pisa, Italy; 3 Istituto Kurz Italia S.r.l., Via Golfo dei Poeti 1/A, 43126 Parma, Italy; c.bender@kurz-italia.com

**Keywords:** arbuscular mycorrhizal fungi, P fertilization, stevia leaf extracts, steviol glycosides, phenol and flavonoid content, antioxidant activities

## Abstract

This work aimed to establish the synergic role of arbuscular mycorrhizal fungi (AMF) symbiosis, phosphorus (P) fertilization and harvest time on the contents of stevia secondary metabolites. Consequently, steviol glycosides (SVglys) concentration and profile, total phenols and flavonoids as well as antioxidant assays, have been assessed in inoculated and no-inoculated plants, grown with or without P supply and collected at different growth stages(69, 89 and 123 days after transplanting).The obtained results suggest that the synthesis of stevia secondary metabolites is induced and/or modulated by all the investigated variability factors. In particular, AMF symbiosis promoted total SVglys content and positively influenced the concentration of some minor compounds (steviolbioside, dulcoside A and rebaudioside B), indicating a clear effect of mycorrhizal inoculation on SVglys biosynthetic pathway. Interestingly, only the mycorrhizal plants were able to synthesize rebaudioside B. In addition, P supply provided the highest levels of total phenols and flavonoids at leaf level, together with the maximum in vitro antioxidant activities (FRAP and ORAC). Finally, the harvest time carried out during the full vegetative phase enhanced the entire composition of the phytocomplex (steviolbioside, dulcoside A, stevioside, rebaudioside A, B, C. total phenols and flavonoids). Moreover, polyphenols and SVglys appeared to be the main contributors to the in vitro antioxidant capacity, while only total phenols mostly contributed to the cellular antioxidant activity (CAA). These findings provide original information about the role played by AMF in association with P supply, in modulating the accumulation of bioactive compounds during stevia growth. At the cultivation level, the control of these preharvest factors, together with the most appropriate harvest time, can be used as tools for improving the nutraceutical value of raw material, with particular attention to its exploitation as functional ingredient for food and dietary supplements and cosmetics.

## 1. Introduction

*Stevia rebaudiana* Bertoni is an herbaceous species, well known for a long time as a source of natural sweeteners, thanks to the presence, in its leaves, of the so-called steviol glycosides (SVglys). These are a group of high-intensity and zero-calorie natural sweeteners, belonging to the ent-kaurenoid diterpene glycosides group [1]. According to the World Health Organization (WHO) recommendations to reduce free sugar intake below 5% of total energy intake, SVglys represent an useful alternative to cope the ongoing demand from the food industry of alternative and “all natural” sugar substitutes, able to reduce the caloric intake of foods and beverages, without compromising their sweetness. Since 2011, the use of highly purified SVglys is approved as a food additive (E960) in the European Union. This European Regulation, together with the growing consumer trends towards natural ingredients and clean label solutions, have increased the stevia sweetener market in Europe. Europe is the second largest sugar substitute market after North America, and geographically segmented into UK, Germany, France, Spain, Russia, Italy and Rest of Europe, where a growing number of companies are manufacturing products containing steviol glycosides. Chemically, SVglys are diterpenoids with an ent-kaurene skeleton, and they differ in number and type of monosaccharide units bound to the common steviol skeleton. To date, more than 34 SVglys have been detected, among which stevioside (Stev) and rebaudioside A (Reb A) are the most abundant. Between them, Reb A shows the best sweet taste, because of the absence of aftertaste. Nonetheless, some minor SVglys are characterized by highly positive taste profile, such as rebaudiosides D and M [2,3]. 

Beyond the presence of SVglys, stevia leaves also represent an interesting source of other valuable bioactive compounds. In fact, within its leaves, considerable amounts of phenols, flavonoids, vitamins and essential oils have been detected [2]. Thanks to the presence of these secondary metabolites, stevia leaf extracts can exert beneficial effects on human health through their antioxidant and biological activities [1,4,5]. All these aspects are becoming increasingly important, especially following the recent approval of stevia leaves as “Traditional Food” in the European Union. In fact, starting from June 2017 stevia leaves can be used for herbal, fruit and tea infusions, and the nutraceutical value of the raw material could be an opportunity for the market of stevia dry leaves. 

Genotype, environment and agricultural management strongly affect the composition and profile of entire stevia phytocomplex, deriving from the synergistic combination of all bioactive compounds within its leaves. In particular, several pre-harvest factors such as fertilization, water management, light, planting time and geometry and crop age are reported to significantly influence both SVglys and secondary metabolites [6,7,8,9,10]. In order to obtain very high-quality raw material, is of pivotal importance to explore the phytochemical variability depending on agro-techniques and pedo-climatic conditions of cultivation site. Moreover, plants can use secondary metabolites as signals for communication between plants and symbiotic microorganisms [11], including beneficial soil microorganisms such as AMF. In turn, AMF, besides to promote plant nutrition and growth, can affect the biosynthesis of plant secondary metabolites with health-promoting activity [12]. AMF are ubiquitous obligate symbiotic microorganisms belonging to the phylum of Glomeromycota [13], that form a mutualistic symbiotic association with most land plants. Biofertilizers, such as AMF, represent a tool for improving host plant productivity and health and, consequently, the overall quality of the raw material. It has been observed that AMF symbiosis can determined a series of beneficial effects on host plant, such as an improvement in plant phosphorus (P) uptake [14], and an increase of secondary metabolite contents in different fruits and vegetable [15]. Anyway, only few studies have specifically investigated the influence of AMF and P fertilization on stevia response and phytochemical profile. Our previous findings [16] demonstrated that, in stevia, AMF symbiosis was able to improve plant P uptake and plant P use efficiency, as well as SVglys yield. Mandal et al. [17,18] have shown that AMF can increase both Stev and Reb A concentration. Nevertheless, to the best of our knowledge, no evidence has been reported about the role of mycorrhizal inoculation, in combination with P fertilization, on total phenols and flavonoids content as well as on antioxidant activity in stevia. 

Thus, the aim of the current study was to define, for the first time, the synergic role of AMF symbiosis, P fertilization and harvest time in affecting stevia main secondary metabolites. To reach this goal, SVglys concentration and profile, total phenols and flavonoids as well as antioxidant capacity -by in vitro and cell-based assays- have been evaluated in inoculated and no-inoculated stevia plants, grown with or without P supply and collected at different growth stages, shedding light on the accumulative dynamics of bioactive compounds in stevia leaves. The present study wish to provide useful information about (i) the understanding of plant responses to the use of AMF in association with P supply and how they act in the biosynthesis/accumulation of the main bioactive compounds and their related chemical and biological antioxidant properties; (ii) the most appropriate harvest time to obtain the highest bioactive compound accumulation.

## 2. Results

### 2.1. Secondary Metabolites and Antioxidant Activities

Data on phytochemical evaluation and antioxidant activities are reported in Table 1. Total phenolic content (TPC) was significantly affected by IxPxDAT interaction as well as by all single variability factors and the reciprocal interaction, exception given for PxDAT interaction. On the other hand, total flavonoids were significantly affected by all the variability factors and their mutualistic interactions, exception given for IxPxDAT interaction.

Regarding inoculation effects, it was observed that non mycorrhizal plants (NMP) showed the higher values of both total phenols and flavonoids, compared to mycorrhizal ones (MP). At the same time, also P supply positively influenced these secondary metabolites. For the harvest time, it was interesting to note that TPC significantly decreased passing from the vegetative phase to the reproductive one, with the lowest value at 123 DAT. An opposite trend was observed for total flavonoids content (TFC), being the highest at 123 DAT (Table 1).

In vitro antioxidant activities measured by FRAP, ORAC and DPPH assays were significantly affected by the investigated variability factors, even if in different way (Table 1). For FRAP and ORAC, significant effects were observed for all the variability factors and the reciprocal interaction, exception given for inoculation effect on FRAP values. Conversely, for this parameter, a clear effect of P supply and harvest time was detected, with the highest antioxidant capacity in plants grown with 25 P rate and collected at the vegetative growth stages (69 and 89 DAT). Similarly, ORAC values were maximum with P supply, but the lowest values were recorded in the first sampling (Table 1). Concerning to DPPH, only sampling date and DATxI interaction significantly affected this parameter. In particular, an increase of the antiradical activity was detected from 69 to 89 DAT. This increase was followed by a reduction, passing to the reproductive phase (Table 1). Cellular antioxidant activity, measured by the CAA assay, significantly varied depending on all variability factors and their interactions, except for IxP and IxPxDAT interactions. The highest cellular activity was reached in non-inoculated plants and without P supply (Table 1). Similarly, to that observed for ORAC and DPPH assays, plant collected at 89 DAT showed the highest cellular antioxidant activity.

Finally, total SVglys content was significantly affected by all the investigated variability factors and their interactions, except for P supply that did not modify the amount of these compounds. Interestingly, AMF inoculation significantly improved the total SVglys in stevia leaves and, as expected, the maximum level of these metabolites was achieved during the maximum vegetative growth (89 DAT).

**Figure 1:**
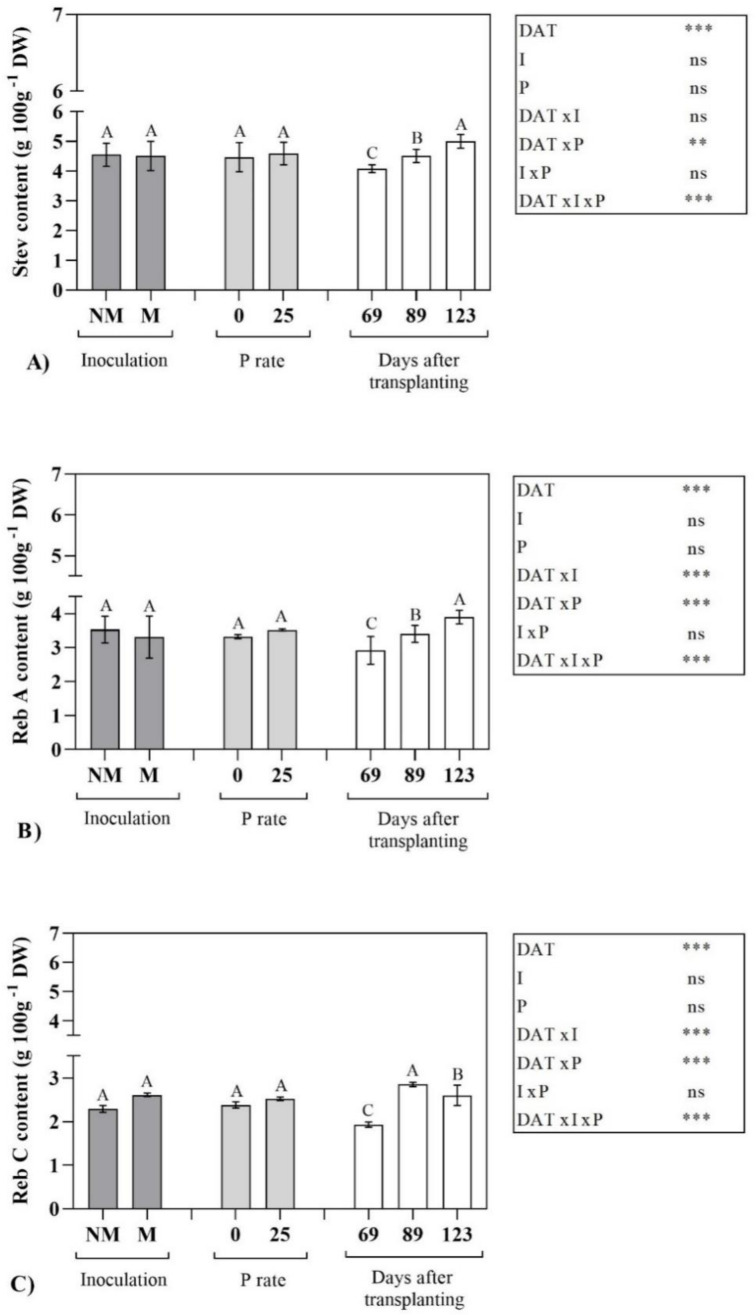
Effect of inoculation (I), P rate (P) and days after transplanting (DAT) on the content (g 100g^−1^ DW) of Stevioside (**A**), Rebaudioside A (**B**) and Rebaudioside C (**C**) in *Stevia rebaudiana* Bert. leaf extracts. Means followed by the same letters are not significantly different at *p* < 0.05 according to L.S.D. Significance of variability factors (I, P and DAT) and their interaction are also reported, as follows: ns, not significant; **, significant at *p* ≤ 0.01; ***, significant at *p* ≤ 0.001 level. Stev, stevioside; Reb A, rebaudioside A; Reb C, rebaudioside C.

**Figure 2:**
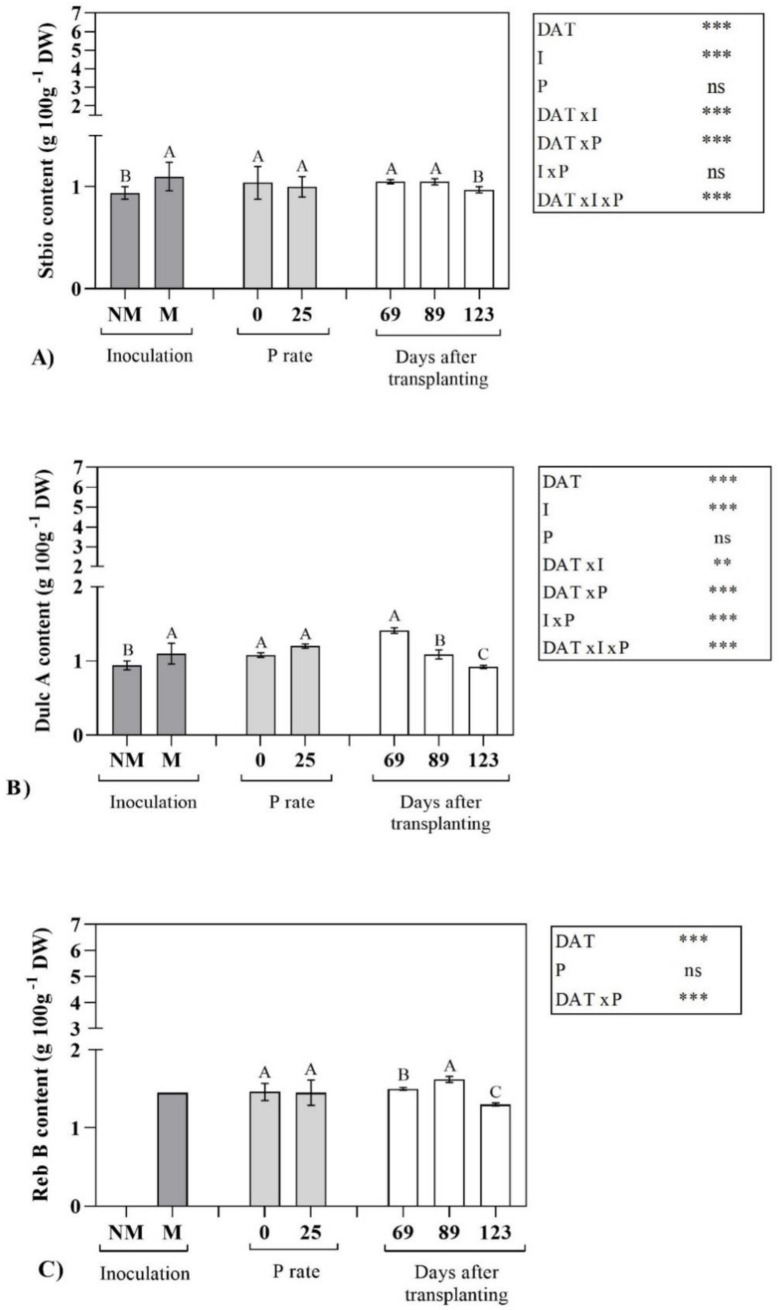
Effect of inoculation (I), P rate (P) and days after transplanting (DAT) on the content (g 100g^−1^ DW) of Steviolbioside (**A**) and Dulcoside A (**B**) in *Stevia rebaudiana* Bert. leaf extracts. For Rebaudioside B (**C**), only the effects of P rate and Days after transplanting have been shown. Means followed by the same letters are not significantly different at *p* < 0.05 according to L.S.D. Significance of variability factors (I, P and DAT) and their interaction are also reported, as follows: ns, not significant; **, significant at *p* ≤ 0.01; ***, significant at *p* ≤ 0.001 level. Stbio, steviolbioside; Dulc A, dulcoside A; Reb B, rebaudioside B.

In particular, Reb B was not detectable in the leaves of non-mycorrhizal plants, to suggest that its biosynthesis carried out only in presence of AMF symbiosis. As already observed for the total SVglys, P supply had no significant effect and, therefore, no differences were observed in plants grown with or without P (Figure 1 and Figure 2). 

### 2.2. Steviol Glycoside Profile

In Figure 1 and Figure 2, SVglys profile and related statistical significance have been reported. As general trend, DATxIxP significantly affected each identified compound, while some differences among the compounds were observed depending on the interaction considered.

It is interesting to note that AMF inoculation had no effect on the content of the main (or most abundant) steviol glycosides such as stevioside (Stev), rebaudioside A (Reb A) and rebaudioside C (Reb C), that did not differ between inoculated and non-inoculated plants (Figure 1). On the contrary, steviolbioside (Stbio), dulcoside A (Dulc A) and rebaudioside B (Reb B) showed a significant enhancement due to AMF symbiosis (Figure 2). Regarding to harvest time, differently than that observed for the total SVglys, the content of Stev and Reb A significantly increased from the vegetative phase to the beginning of the reproductive one, with the highest values at 123 DAT. Otherwise, accordingly to the total SVglys behavior, Reb C and Reb B were maximum at 89 DAT. Stbio showed the highest levels during the whole vegetative stage (69 and 89 DAT) and then its content significantly decreased at 123 DAT. Finally, Dulc A showed an opposite trend than Stev and Reb A, with the highest content in the 1st sampling (Figure 2).

### 2.3. Effects of Bioactive Compounds on Antioxidant Activities

In order to determine the contribution of specific secondary metabolites to the antioxidant activities of the leaf stevia extracts, an analysis of linear regression was carried out (Table 2). TPC was significantly and directly correlated with both FRAP and CAA and negatively with TFC, but no correlations were found with ORAC, DPPH and total SVglys. Otherwise, TFC was significantly and positively correlated with ORAC and total SVglys, while these compounds were inversely correlated with TPC and CAA. No correlations have been found between TFC and FRAP and between TFC and DPPH. Furthermore, a positive and direct correlation between SVglys and FRAP was observed.

Finally, the different assays used for antioxidant activity evaluation correlated with each other, exception given for ORAC and CAA. In particular, FRAP positively correlated with ORAC and CAA, while DPPH negatively correlated with the other assays. To evaluate the reliability of the antioxidant activity results obtained via traditional FRAP, ORAC and CAA assays, we determined the specific antioxidant capacity (SAC) (Table 3). TPC positively correlated with FRAP and CAA, showing SAC values of 7.13 µmol TE/mg GAE and 0.130 µmol QE/mg GAE, respectively. At the same time, also SVglys significantly and positively correlated with FRAP, with a SAC equal to 4.07 µmol TE/mg DW. This result is very interesting and suggests that TPC had major effectiveness in determining antioxidant activity measured by FRAP assay, than SVglys.

Finally, only TFC showed positive relationship with ORAC, exhibiting a SAC of 26.04 µmol TE/mg CE.

## 3. Discussion

### 3.1. The role of Variability Factors on Stevia Bioactive Compounds

In the present study, the role of P fertilization, AMF symbiosis and harvest time on the concentration of bioactive compounds and antioxidant capacity of stevia leaves has been defined, with the aim to extend current knowledge about these issues. The obtained results indicated that P supply provided the best phytochemical content, in terms of total phenols and flavonoids, together with the maximum in vitro antioxidant activities (FRAP and ORAC). It is known that plants require relatively large amount of phosphorus for the biosynthesis of primary and secondary metabolites, since P has essential functions as a constituent of nucleic acids and phospholipids and plays a key role in the energy metabolism of cells [19,20].

Similarly to our results, Nell et al. [20] found, in *Salvia officinalis* L., high correlations between P supply and total phenolic concentrations and between P and rosmarinic acid, to indicate that P supply was able to determine the highest content of total phenols and rosmarinic acid.

Differently than that observed for P, in AMF stevia plants, the biosynthesis of secondary metabolites seemed to be differently regulated. In fact, only total SVglys content and profile were enhanced by AMF symbiosis. In particular, the mycorrhizal inoculation in plants grown in absence of P supply promoted the accumulation of SVglys. In addition, no significant differences for the main SVglys (Stev, Reb A and Reb C) have been observed, while mycorrhizal inoculation would seem to influence the concentration of the minor SVglys, such as Stbio, Dulc A and Reb B.

In fact, Stbio and Dulc A contents were higher in mycorrhizal plant respect to non-mycorrhizal ones. It was interesting to observe that only the mycorrhizal plants were able to synthesize Reb B. These observations indicate a clear influence of mycorrhizal inoculation on the SVglys biosynthetic pathway. At this regard, Mandal et al. [18] showed transcription up-regulation of genes involved in SVglys biosynthetic pathway (MEP) in stevia mycorrhizal plants, respect to non-mycorrhizal ones. Nevertheless, these authors only take into account Stev and Reb A and no information was reported on other SVglys. Moreover, they did not consider the fact that, after the formation of steviol through MEP, a series of glycosylations takes place in the cytosol catalyzed by more than a dozen cytosolic UDP-dependent glycosyltransferases (UGTs),and that two of these showed 98% homology [21]. In fact, among UGTs enzymes, the UGT76G1 synthesizes Reb A from Stev, as well as it is suspected to catalyses the formation to Reb C from Dulc A [21], furthermore, it is further involved in the formation of Reb B using Stbio as precursor [22]. In the present study, Stbio, one of the first compounds to be synthesized in the SVglys metabolic pathway, was found in major concentration in M plants, suggesting that the mycorrhizal inoculation could stimulate the SVglys biosynthesis. The highest concentration of Stbio in M plants could be responsible for the synthesis of Reb B in such plants, since Reb B is directly synthesized from Stbio. Respect to Mandal et al. [18] observations the process seems to be more complex and dynamic, thus the increase in transcription of MEP following mycorrhizal symbiosis must be further investigated.

Our study underlined a clear effect of harvest time in defining the entire stevia phytocomplex, for example to maximize the content of the individual compounds (exception given for flavonoids) and to improve the antioxidant activities, both in vitro and at cellular level, the harvesting during the full vegetative plant development showed to be more appropriate. This behaviour was already observed in previous studies in relation to steviol glycosides [7,9,23], confirming that, in stevia, plant development is a primary factor in influencing the biosynthetic pathway of these secondary metabolites. In fact, at each sampling date, SVglys proportion significantly changed with an increase of Stev and Reb A content, passing from the vegetative to the reproductive phase, while, on the contrary, Reb C, Stbio, Dulc A and Reb B significantly decreased in the last harvest. In addition, our findings pointed out that, in stevia, the plant development plays a key role also in defining the accumulation of total phenols and flavonoids and their related antioxidant activity. Therefore, the identification of the optimal harvest time, able of maximizing all bioactive compounds and, consequently, the nutraceutical properties of raw material, is of paramount importance for the growth of stevia market.

### 3.2. The Role of Variability Factors on Antioxidant Activities

Beyond its sweetening power, stevia leaves represent a good source of bioactives, due to the presence of phenols and flavonoids. Currently, the demand for these bioactive constituents and their derivatives has increased because they are natural, eco-friendly and generally recognized as safe products [24]. Therefore, they represent valuable functional ingredients for the pharmaceutical, food and feed industries. In particular, in the food industry, they can find applications as natural preservatives, thanks to their capacity to retard oxidative degradation of lipids and to improve the shelf life and the qualitative and nutritional characteristics of foods and beverages [25]. These compounds, in fact, are exogenous natural antioxidants involved in the prevention of oxidative stress in humans, thanks to the presence in their molecules of hydroxyl groups [26,27,28], able to exert antimicrobial, antioxidant, antiparasitic, antifungal, and anti-inflammatory properties. At this regard, we estimated the antioxidant activity of stevia leaves and its variation depending on AMF inoculation, P supply and harvest time, through different in vitro assays and one cellular test. In the evaluation of antioxidant activity of plant extracts, it is of key importance to carry out more than one antioxidant assay [27], since these tests differ for both mechanism of action and target molecules. Overall, our data, confirming previous reports [4,7,29], showed that stevia leaf extracts were characterized by a noticeable antioxidant activity. As reported in Tavarini et al. [9], the IC_50_ values of stevia leaf extracts were lower than that of some commonly used antioxidants, such as BHT. Furthermore, the antioxidant activity of stevia leaves is similar or sometimes higher than that of other most known natural sources of antioxidants, such as green tea, berries and berry products, dried mint leaves, saffron, oregano, dark chocolate [30,31]. The assessment of stevia antioxidant properties continues to be an interesting and useful task, particularly for finding new sources of natural antioxidants, functional food ingredients and nutraceuticals. As observed for the bioactive compounds, also for the antioxidant activities, different behaviors were detected in response to the investigated variability factors. As general trend, the obtained results indicated that AMF symbiosis did not influence the antioxidant activities and the non-mycorrhizal plants showed the higher values in comparison with mycorrhizal ones. On the contrary, P supply was able to improve the antioxidant activities assessed by FRAP and ORAC, but no effects were detected at a cellular level (CAA test). In addition, the harvest time influenced the antioxidant activities, with all different assays applied, with the highest values obtained when the plants were ending their leaf biomass development (89 DAT), accordingly to the individual compounds. Similarly to our study, several reports pointed out the importance of these pre-harvest factors, especially about the role of harvest time in influencing the antioxidant activities indifferent medicinal and aromatic plant species [32,33,34].

### 3.3. Antioxidant Activities and Secondary Metabolites Correlation

With the aim to evaluate the correlations among the antioxidant activities and the secondary metabolites present in stevia leaf extracts, an analysis of linear regression was carried out (Table 2). This allowed us to understand whether antioxidant capacities could be predicted from one assay to another. The results indicated that, thanks their positive correlation with FRAP and ORAC assays, TPC, TFC and SVglys might be the main contributors to these in vitro antioxidant capacities, as previously reported [7,9,35]. Moreover, the positive Pearson’s correlation coefficient observed between total phenolic content and CAA, suggested that these secondary metabolites, contrary to SVglys, could mostly contribute to the intracellular antioxidant activity [29], also in agreement with other studies carried out on different fruits and vegetables [36,37]. This can be understood considering that, on the one hand, purified SVglys (stevioside and rebaudioside A) showed a discrete in vitro antioxidant capacity (measured by FRAP assay), but a low antioxidant activity measured by ORAC [35], even if the extent to which they play a role in the overall response of *S. rebaudiana* leaves to oxidative stress is not yet clear. On the other hand, they did not show an antioxidant capacity at cellular level (CAA assay); probably, due to the size of their chemical structure, not all of them can get through cell membranes [29,38]. Consequently, the lack of a positive correlation between CAA and SVglys can suggest that steviol glycosides can hardly be absorbed by liver cells in vitro, confirming previous reports [35].

Many studies have reported the strong contribution of phenolic compounds to the antioxidant activity, measured through different assays, in several plant matrices and foods [39,40]; this relationship depends on many factors, such as genetic characteristics, maturity stages, and environmental conditions. The different mixture of phenolics can act synergistically or antagonistically to inhibit free radicals and this depends on the specific phenolic profile, which can be qualitative (type of phenolic present) or quantitative (the relative amounts or proportions of phenolics present) [30]. Clearly, the antioxidant activity of a specific phenolic or flavonoid compound is related with the number of available hydroxyl groups present in the chemical structure and the presence of glycosylations on the molecule may decrease its antioxidant activity [39,40,41,42,43]. To evaluate the reliability of the antioxidant activity results obtained via traditional FRAP, ORAC and CAA assays, we determined the specific antioxidant activity (SAC) reporting the antioxidant activity on a phenolic basis, according to Jacobo-Velázquez and Cisneros-Zevallos concept [41], as well as based on total flavonoid (TFC) and SVglys. Reporting the antioxidant activity on structure-activity relationship allows to obtain information about the effectiveness of stevia phenolic compounds to neutralize free radicals, where a higher specific antioxidant capacity means phenolic compounds have a higher capacity to stabilize free radicals [44]. In fact, our results highlighted how total phenols, compared to steviol glycosides, gave a greater contribution to the antioxidant activity in vitro (FRAP method) and in vivo (CAA method). In addition, total flavonoids provided a more important contribution to the antioxidant activity of stevia leaf extract measured by ORAC assay. A negative Pearson’s correlation coefficient was obtained between total flavonoids and in vivo assay (CAA method), suggesting strong relationships among CAA and molecular structure of the specific flavonoids present in the tested extracts [45].

Furthermore, interestingly significant correlation was also found between SVglys and flavonoids, indicating that both types of metabolites are simultaneously involved in stevia response to the combined effect of AMF inoculation, P supply and harvest time. Similar findings have been also observed by Tavarini et al. [9], where a positive correlation between SVglys and flavonoids suggested a possible interrelationship of flavonoids with the metabolic pathway of SVglys. This possible role of flavonoids in the stevioside metabolic pathway deserves more investigations and the study of putative candidate genes involved in the two pathways could be helpful to elucidate these mechanisms. Finally, our correlations indicated that only FRAP assay correlated positively with CAA, but no correlation was found with ORAC assay, this in agreement with previous studies [37,46,47,48].

## 4. Materials and Methods

### 4.1. Chemicals

Trolox (6-hydroxy-2,5,7,8-tetramethylchroman-2-carboxylic acid), fluorescein sodium salt, 2,2′-azobis [2 -methylpropionamide] dihydrochloride (AAPH), quercetin dihydrate, 2′-7′-dichlorodihydrofluorescin diacetate (DCFH-DA), were purchased from Sigma-Aldrich (Milan, Italy). Dulbecco’s Modified Eagle’s Medium (DMEM) high glucose culture media, penicillin, streptomycin, L-glutamine, trypan blue, trypsin, were from culture grade and also purchased from Sigma-Aldrich. Fetal bovine serum (FBS), Dulbecco’s phosphate buffered saline (PBS) without Mg^2+^ and Ca^2+^ and Hank’s balanced salts solution (HBSS) from culture grade were purchased from Euroclone SpA (Milan, Italy). Methanol was purchased from Carlo Erba SpA (Milan, Italy). Water and acetonitrile were obtained from J. T. Baker (Phillipsburg, NJ, USA). A Common Steviol Glycosides Standards Kit (steviolbioside, dulcoside A, stevioside, rebaudioside A, B, and C) was purchased from Chromadex (LGC Standards S.r.L., Milan, Italy). All other chemicals were from analytical high-performance liquid chromatography (HPLC) grade and purchased from common sources. All solvents and water were accurately degassed before being used in the analyses.

### 4.2. Plant Material and Experimental Conditions

The tested stevia genotype, belonging to the germplasm collection of the Department of Agriculture, Food and Environment (DAFE, University of Pisa, Italy), is characterised by high content of rebaudioside C (Reb C). The plants were obtained by stem cuttings as described by Tavarini et al. [16], to avoid plant genetic variability in terms of both morphological and phytochemical traits. A pot trial was carried out in open air conditions at the Experimental Centre of DAFE located in Central Italy (Tuscany, Pisa, 43° 40′ N; 10° 19′ E), starting from 5 May 2015 until 8 September 2015. The area is characterized by a typically Mediterranean climate. Air temperatures and rainfall were recorded through weather station close to the experimental site. During the trial season mean maximum and minimum temperatures were 28.8 °C and 15.8 °C, respectively, and total rainfall was 283.8 mm. The substrate was a mixture of 9/10 sandy-loam soil (sand 75%; silt 22%; clay 3%; organic matter 1.5%; pH 8.1; total nitrogen 0.6 g kg^−1^; available phosphorus 11.9 mg kg^−1^; exchangeable potassium 107.1 mg kg^−1^) and 1/10 peat-based growing media (Valcofert S.r.l, Empoli, Italy). Stevia plants were exposed to different treatments, consisting in different P doses (0 and 25 mg P_2_O_5_ kg^−1^ of soil, namely 0P and 25P) with (MP) or without (NMP) mycorrhizal inoculation. The plants were maintained under optimal nitrogen (0.25 g N pot^−1^ as ammonium nitrate) and water conditions. No pests or diseases were observed during the experiment. A randomized block design with two treatments (mycorrhizal inoculation and P fertilization), each replicated ten times, was adopted. Three different destructive samplings were carried out at 69, 89 and 123 days after transplanting (DAT). According to the Biologische Bundesanstalt, Bundessortenamt and Chemical industry (BBCH) phenological growth stages proposed by Le Bihan et al. [49] for stevia, we have defined the three harvest times as follows: 69 DAT corresponds to Stage 43 = 30% of harvested part of the plant is developed; 89 DAT corresponds to Stage 49 = ending of leaf biomass development; about 90% of final leaf biomass is developed; 123 DAT corresponds to Stage 55 = 50% of apex leaves are differentiated and present inflorescence, but flower buds are still closed. Leaves of each single replicate were air-dried in a ventilated oven from 30 to 40 °C until constant weight. Dry leaves were minced to a fine powder by a laboratory mill (Grindomix GM 200, Retsch, Retsch Italia, Pedrengo (BG), Italy) and stored until the subsequent analyses.

### 4.3. AMF Inoculum

*Rhizoglomus irregulare* (N.C. Schenck & G.S. Sm.) Sieverd, G.A. Silva & Oehl, (syn. *Rhizophagus irregularis* C. Walker & A. Schüssler, formerly known as *Glomus intraradices*) isolate IMA6 derived from the International Microbial Archive (IMA) collection of the Department of Agriculture, Food and Environment (DAFE) of the University of Pisa and it was obtained from pot cultures of *Trifolium alexandrinum* as host plants and, after accurate identification, it was stored at IMA collection. This mycorrhizal fungus, provided by the laboratory of Microbiology of DAFE, was used as inoculum, following the procedure described by Tavarini et al. [16].

### 4.4. Steviol glycosides determination

Extraction procedures and SVglys determination were assessed according to Woelwer-Rieck et al. [50] with slight modifications. A Luna HILIC 200A, 5μm, 250 mm × 4.6 mm (Phenomenex S.R.L. Inc., Bologna, Italy) column in conjunction with the corresponding guard column (4.0 × 3.0 mm) was used, in a HPLC system (Jasco PU980) (JASCO Europe S.r.l., Cremella, Italy) coupled with a UV-visible wavelength detector. The following HPLC operating conditions were used: an isocratic mobile phase, acetonitrile/water (80:20), a flow rate of 0.68 mL/min, and a run time of 20 min. Detection was at 210 nm at ambient temperature. Chromatograms were acquired online, and data were collected via a Jasco interface (Hercules 2000 interface chromatography). Steviol glycosides quantification was performed using authentic standards through calibration curves (0.05–0.5 g L^−1^) obtained from standard mixtures containing steviolbioside, dulcoside A, stevioside, rebaudioside A, B, and C.

### 4.5. Preparation of Extract

Stevia extracts were prepared by infusion of powdered dry leaves in 100 °C distilled water at 10 g L^−1^ final concentration and sonicated for 10 min. The resulting extracts were centrifuged for 10 min at 3000 rpm, the supernatants were sterile filtered through a sterile 0.2 µm syringe filter and immediately analysed or stored at −80 °C before use for up to a week.

### 4.6. Analysis of Total Phenols and Flavonoids

Total phenols were determined using the Folin-Ciocalteu method according to Singleton et al. [51]. This method involved the reduction of Folin-Ciocalteu reagent by phenolic compounds, with a blue complex formation determined at 765 nm by UV-Vis spectrophotometer (Varian Cary 1E, Palo Alto, CA, USA). The results were expressed as mg gallic acid equivalent (GAE) per gram of stevia leaves on dry basis. Total flavonoids were determined using the method described by Jia et al. [52] based on the reaction flavonoids-aluminum which allowed a pink complex formation measured at 510 nm using a UV-Vis spectrophotometer (Varian Cary 1E, Palo Alto, CA, USA). The results were expressed as mg catechin equivalent (CE) per gram of stevia leaves on dry basis.

### 4.7. Ferric Reducing Antioxidant Power (FRAP) Assay

The determination of ferric reducing antioxidant power of stevia leaf extracts was made according to the method described by Benzie and Strain [53] and it is based on the ability of the antioxidant compounds to reduce Fe^3+^ to Fe^2+^ which, in the presence of TPTZ (2,4,6-tris(2-pyridyl)-s-triazine), led to a blue complex formation (Fe^2+^-TPTZ) measured at 593 nm using a UV-Vis spectrophotometer (Varian Cary 1E, Palo Alto, CA, USA). Trolox was used as standard for constructing calibration curves and the values were expressed as mmol Trolox equivalent (TE) per gram of stevia leaves on dry basis.

### 4.8. Oxygen Radical Absorbance Capacity (ORAC) Assay

ROS scavenging capacities of stevia leaves were assessed by the ORAC test [54,55] with minor modifications. Briefly, serial dilutions were prepared fresh in phosphate buffer (75 mM, pH 7.4) and analysed in duplicate wells in a 96-well black plate containing a 10 nM solution of fluorescein. The plate was incubated for 30 min at 37 °C, the background signal was determined and a 240mM solution of AAPH was added into each well. Fluorescence measurements (Ex. 485 nm, Em. 520 nm) were recorded for an hour at 37 °C (Fluostar Optima, BMG Labtech, Offenburg, Germany). Raw data were analysed with MARS 2.0 Optima data analysis software (BMG Labtech, Offenburg, Germany); the integrated area under the fluorescence curve (AUC) was calculated for each sample and corrected with blanks AUC. Trolox equivalents (TE) were calculated based on the reference AUCs and the ORAC values expressed as mmol TE per gram of stevia leaves on dry basis.

### 4.9. Cellular Antioxidant Activity (CAA) Assay

HepG2 cells were grown in DMEM high glucose medium supplemented with 10% heat inactivated FBS, 2mM l-glutamine, 50 μg mL^−1^ penicillin and 50 μg mL^−1^ streptomycin and maintained at 37 °C under a humidified atmosphere of 5% CO_2_. At each experiment the viability was estimated by trypan blue exclusion, while the toxicity of the stevia extracts was assessed by measuring the cytosolic lactate dehydrogenase activity as previously reported [29]. The CAA assay was conducted according to the protocol of Wolfe and Liu [46], with minor modifications. Cells were seeded in a 96-well black plate with transparent bottom (Greiner Bio-One GmbH, Frickenhausen, Germany) and incubated at 37 °C and 5% CO_2_ up to 24 h or until 80% of confluence was reached. Stevia extracts testing dilutions, standard dilutions, and control untreated cells were prepared fresh in DMEM 25 mM d-glucose and analysed in five replicate wells containing a 25µM solution of DCFH-DA. The plate was incubated for 60 min at 37 °C before washing out twice the unabsorbed molecules with PBS. The background signal was determined (Fluostar Optima reader, BMG Labtech, Offenburg, Germany), before a 600 µM solution of AAPH in HBSS was added into each well. Fluorescence (Em. 485 nm, Ex. 540 nm) was recorded immediately and for an hour at 37 °C and the raw data were analysed with MARS 2.0 Optima software (BMG Labtech). The AUC was calculated for each sample and corrected with blanks AUC. Quercetin equivalents (QE) were calculated based on the reference standard curves and CAA values were expressed as μmol QE per gram of stevia leaves on dry basis.

### 4.10. Free Radical-Scavenging Assay

The free radical-scavenging activity of stevia water leaf extracts was evaluated by the DPPH (1,1-diphenyl-2-picryl-hydrazil radical) free radical assay according to the method described by Brand-Williams et al. [56]. The assay is based on the reducing activity of the antioxidant molecules against the DPPH radical characterized by a purple red colour. The extent of disappearance of DPPH is directly proportional to the amount of antioxidant present in the reaction measured at 515 nm using a UV-Vis spectrophotometer (Varian Cary 1E, Palo Alto, CA, USA). Trolox was used as standard for constructing calibration curves and the values were expressed as mmol TE per gram of stevia leaves on dry basis.

### 4.11. Specific Antioxidant Capacity (SAC)

The specific antioxidant capacity (SAC) of bioactive compounds present in stevia leaf extracts was evaluated according to Jacobo-Velázquez and Cisneros-Zevallos [41] and Vizzotto et al. [44]. SAC has been evaluated as the ratio of total antioxidant capacity per total soluble phenolics (expressed as µmol of Trolox equivalents per mg of gallic acid), as well as per total flavonoids (expressed as µmol of Trolox equivalents per mg of catechin) and per total steviol glycosides (expressed as µmol of Trolox equivalents per mg of dry leaves). The specific antioxidant capacity provided information about the effectiveness of polyphenols and SVglys to neutralize free radicals.

### 4.12. Statistical Analysis

All data were subjected to analysis of variance (ANOVA) using GraphPad Prism version 8.0.2 (GraphPad Software, Inc., La Jolla, CA, USA). Phenolic and flavonoid contents, antioxidant capacities and total SVglys were analyzed using a three-way ANOVA completely randomized to estimate the variance components of days after transplanting (DAT), phosphorus (P), mycorrhizal inoculum (I) and their reciprocal interactions (I x P, I x DAT, P x DAT, I x P x DAT). One-way ANOVA, t-test and three-way ANOVA were used to estimate significant differences between obtained values for each SVgly compound (Stev, Reb A, Reb B, Reb C, Stbio and Dulc A).Means were separated on the basis of least significant difference (LSD) only when the ANOVA F test showed significance between the 0.05 and 0.001 probability level. Finally, linear regression analyses using GraphPad Prism version 8.0.2 (GraphPad Software, Inc., La Jolla, CA, USA) were performed to evaluate the relationships among total phenols, total flavonoids, antioxidant capacities and total SVglys.

## 5. Conclusions

This study adds knowledge about the effects of AMF symbiosis, P supply and harvest time on the content of secondary metabolites in stevia leaf extracts. The obtained results suggest that the synthesis and accumulation of secondary metabolites in stevia are induced and/or modulated by the investigated variability factors simultaneously. The use of AMF symbiosis did not increase total polyphenols, while a positive influence on total SVglys content and Stbio, Dulc A and Reb B has been showed. Further investigations will need to be carried out for understanding the mechanisms involved in accumulation and biosynthesis of these minor compounds under mycorrhizal inoculation. However, a P supply led to an increase of total phenols and flavonoids as well as of in vitro and cell-based antioxidant activities, probably by affecting the phenylpropanoid pathway. The harvest time strongly alters the health-promoting compounds in stevia, and, for this reason, the identification of the most appropriate plant developmental stage is essential to obtain the highest content of bioactive compounds. The good linear correlations found among phenols, flavonoids and SVglys and FRAP and ORAC, suggest that these compounds could be used as an indicator of in vitro antioxidant properties of stevia leaf extracts. At the same time, phenolic compounds could be also used as an indicator of the in vivo antioxidant activity determined by CAA assay. In conclusion, the presented work demonstrated that stevia leaf extracts contained significant amounts of biologically active compounds and might be used as functional ingredients for food, dietary supplements and cosmetics. This could open new perspectives towards the traditional use of whole stevia leaves, with consequent benefits for small farmers.

## Figures and Tables

**Table 1:** Results of three-factorial analysis of variance (ANOVA) for secondary metabolites (total phenols, flavonoids and steviol glycosides) and antioxidant activities of stevia leaf extracts.

Factor	Factor Level	TPC (mg GAE/g DW)	TFC (mg CE/g DW)	FRAP (mmol TE/g DW)	ORAC (µmol TE/g DW)	CAA (µmol QE/g DW)	DPPH (mmol TE/g DW)	Total SVglys (g/100g DW)
Main Effects								
Mychorizzal inoculation (I)	NMP	81.91 ± 3.00 a	71.13 ± 2.39 a	0.560 ± 0.02 a	2231.19 ± 106.2 a	11.28 ± 0.77 a	0.161 ± 0.002 a	12.95 ± 0.30 b
	MP	74.00 ± 3.77 b	68.92 ± 0.99 b	0.553 ± 0.03 a	1417.01 ± 90.30 b	9.14 ± 0.73 b	0.163 ± 0.002 a	14.37 ± 0.57 a
Phosphorus fertilization (P)	0P	74.88 ± 3.20 b	68.29 ± 2.10 b	0.532 ± 0.03 b	1593.51 ± 113.5 b	10.67 ± 0.77 a	0.161 ± 0.001 a	13.79 ± 1.66 a
	25P	81.04 ± 3.72 a	71.76 ± 1.44 a	0.581 ± 0.02 a	2054.67 ± 139.9 a	9.71 ± 0.78 b	0.162 ± 0.002 a	13.52 ± 0.16 a
Days after transplanting (DAT)	69 DAT	87.06 ± 1.30 a	63.95 ± 1.68 c	0.591 ± 0.02 a	1636.41 ± 183.2 c	10.37 ± 1.03 b	0.155 ± 0.001 c	12.18 ± 0.19 c
	89 DAT	87.64 ± 1.38 a	69.76 ± 0.90 b	0.609 ± 0.03 a	1953.47 ± 75.21 a	12.36 ± 0.33 a	0.168 ± 0.002 a	14.80 ± 0.80 a
	123 DAT	59.17 ± 1.89 b	76.37 ± 1.51 a	0.470 ± 0.01 b	1882.41 ± 129.4 b	7.66 ± 0.26 c	0.162 ± 0.002 b	14.00 ± 0.24 b
***Significance***								
	I	***	*	ns	***	***	ns	***
	P	***	***	***	***	*	ns	ns
	DAT	***	***	***	***	***	***	***
	I x P	*	***	***	***	ns	ns	**
	I x DAT	***	***	***	***	***	*	***
	P x DAT	ns	***	***	***	***	ns	***
	I x P x DAT	**	ns	***	***	ns	ns	***

Data were evaluated via three-way ANOVA, factors: mycorrhizal inoculation (I), phosphorus rate (P) and days after transplanting (DAT). Identical letters indicate that values do not differ significantly at *p* < 0.05 according to LSD. Asterisks (*) indicate significantly influential factors as follows: ns, not significant; **, significant at *p* ≤ 0.01; ***, significant at *p* ≤ 0.001 level. TPC, total phenolic content; TFC, total flavonoid content; FRAP, ferric reducing antioxidant power; ORAC, oxygen radical absorbance capacity; CAA, cellular antioxidant activity; DPPH, 1,1-diphenyl-2-picrylhydrazyl; total steviol glycosides (Total SVglys).

**Table 2:** Linear regression and Pearson coefficient (r^2^) among secondary metabolites (TPC, TFC and total SVglys) and antioxidant activity assays (FRAP, ORAC, CAA and DPPH).

Assay	TPC	TFC	FRAP	ORAC	CAA	DPPH	Total SVglys
**TPC**	1	0.110 * y= −0.156x + 81.53	0.268 ** y= 0.004x + 0.260	0.050 n.s.	0.187 * y= 0.097x + 2.638	0.038 n.s.	0.035 n.s.
**TFC**	0.127 * y= −0.706x + 127.8	1	0.003 n.s.	0.169 * y = 32.51x − 449.9	0.244 ** y = −0.228x + 26.08	0.014 n.s.	0.226 * y = 0.134x + 4.331
**FRAP**	0.268 ** y = 74.24x + 37.71	0.004 n.s.	1	0.230 ** y = 2695x + 362.8	0.202 * y = 14.21x + 2.460	0,141 * y = −5.805x + 1,483	0.212 * y = 8.847x + 8.868
**ORAC**	0.044 n.s.	0.169 * y = 0.005x + 60.23	0.230 * y = 8.55 x 10^-4^ + 0.386	1	0.065 n.s.	0,1767 * y = −36485x + 7739	0.013 n.s.
**CAA**	0.187 * y = 1.951x + 58.47	0.244 ** y = −1.063x + 80.26	0.202 * y = 0.014x + 0.399	0.065 n.s.	1	0,181 * y = −207.7x + 43,84	0.008 n.s.
**DPPH**	0.038 n.s.	0.014 n.s.	0,141 * y = -5.805x + 1.483	0,1767 * y = −36485x + 7739	0,181 * y = −207.7x + 43.84	1	0.001 n.s.
**Total SVglys**	0.035 n.s.	0.226 * y = 1.687x + 46.69	0.212 * y = 0.024x + 0.214	0.013 n.s.	0.007 n.s.	0.001 n.s.	1

Significance was as follows: n.s. not significant; * significant at *p* ≤ 0.05 level; **, significant at *p* ≤ 0.01. TPC, total phenolic content; TFC, total flavonoid content; FRAP, ferric reducing antioxidant power; ORAC, oxygen radical absorbance capacity; CAA, cellular antioxidant activity; DPPH, 1,1-diphenyl-2-picrylhydrazyl; total steviol glycosides (Total SVglys).

**Table 3:** Specific antioxidant activity measured as ratio between antioxidant activities (FRAP, ORAC and CAA) and secondary metabolites (TPC, TFC and SVglys) in stevia leaf extracts on the basis of significant and positive correlations.

	Specific Antioxidant Capacity
**FRAP/TPC** (µmol TE/mg GAE)	7.13
**CAA/TPC** (µmol QE/mg GAE)	0.130
**ORAC/TFC** (µmol TE/mg CE)	26.04
**FRAP/SVglys** (µmol TE/mg DW)	4.07

TPC, total phenolic content; TFC, total flavonoid content; total steviol glycosides (Total SVglys); FRAP, ferric reducing antioxidant power; ORAC, oxygen radical absorbance capacity; CAA, cellular antioxidant activity.
